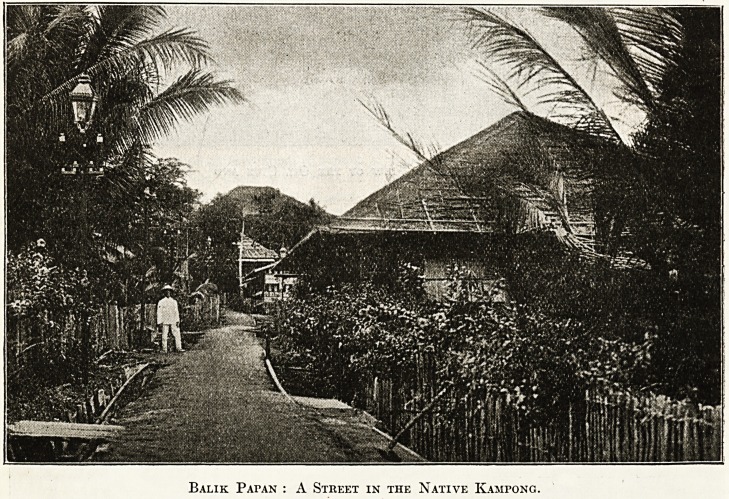# In an East Borneo Hospital.—I

**Published:** 1912-09-14

**Authors:** 


### IN AN EAST BORNEO HOSPITAL.—I.

(BY OUR SPECIAL COMMISSIONER.)

Balik Papan, in the realm of the Sultan of Koetei, on the shores of Dyakland, where marriage with a deceased wife's sister is a crime only slightly less execrable than parricide, is eminently a modern city. City one writes advisedly, for it is not merely a settlement. It is a vast centre of lights burning for ten miles along an esplanade that at night looks as imposing as Blackpool or Havre. In the daytime it resolves itself into a factory town where Industry and Nature seem to be struggling for the mastery. Industry has drained that mosquito-haunted swamp, and built on it a wax factory that produces 12,000 tons of the finest wax on the market. Industry has created that sulphuric-acid factory which turns out eight tons of fuming acid per day; that pier, that electric plant, that foundry where Malay and Chinese coolies weld iron to iron by means of the oxyhydrogen blowpipe. And you see Nature in the jungle that slopes down to the sea and almost threatens to overwhelm the oil refineries. Balik

Balik Papan: General View of the Oil City and Harbour.

Balik Papan: A Street in the Chinese Quarter.

owes its prosperity and its rapid growth to oil. Years ago—but the story has little to do with the hospital. Now the Dutch Petroleum Company lords it here in a wise, paternal fashion that evokes admiration, and that notwithstanding the instinctive prejudice which the visitor may have against coolie labour, " always abused and always a crime," as a French statesman called it. It is the company that has built the new hospital and that has done so much to improve the sanitation of the place. And it is oil that has made the company, and oil, therefore, that is primarily responsible for the hospital.

Early in the forenoon I strolled along the sea road, with the water, a pure malachite, on one side, and the jungle, a tangle of dark beryl and deep emerald, mosaiced by purples where the big rasamala trees stood out in the sun-glare, on the other, to visit Dr. Horst, the genial enthusiastic chief of the establishment. The hospital stands on a slight rise facing the bay, with the jungle lying behind. As it is newly built it has a young, colonial-like look. The hibiscus and oleanders have not grown in its gardens yet; the cheniara trees are young, and the palm trees behind only high enough to shade the low roofs of the pavilions. It stands on a good site, sandy with rocky subsoil, on a slight slope that drains to the sea. The arrangement is on the pavilion system, each pavilion being built of brick and wood, roofed in by hard wood tiles, which are impregnated so as to resist the attacks of insects. Proper precautions are taken to guard against damp—a matter which is one of great concern and difficulty in most tropical hospitals. Balik is almost equatorial. Its average humidity is very high and its average daily difference of temperature is merely a few degrees Centigrade. By proper drainage of the swamps the climatic conditions have been much improved, but a great deal remains to be done. The pavilions are for the most part arranged longitudinally—that is, with their long axes S.E. and N.W., allowing for the maximum amount of light, the inclination E. and W. being slightly less than in Europe. In the morning sunlight the pavilions, neat in their green and light brown, look pretty against the jungle background. The front pavilion is given over to the out-patient department, the pharmacy, and the office. Here Dr. Horst attends every morning to see numerous coolies who come for slight ailments. These he examines and turns over to his native dressers or mandoers, who are readily trained to the work and make really efficient and first-class assistants. At the time of my visit I found the superintendent pondering over something that had gone wrong in the elaborate new micro-photographic apparatus, and which he could not readjust. He left it in despair to show me over the institution, and when we returned we found that one of the mandoers, who had been studying the instrument, had completed the readjustment with excellent success. All the blood examinations and the routine examinations for ankylostoma ova are carried out by natives who have been trained by Dr. Horst, and whose laboratory methods are strikingly precise and neat. The Malay undoubtedly has the artistic instinct strongly developed, and seems to take to microscopic work as a duck takes to water. The slides were all excellently done and the " spotting " was, from a diagnostic point of view, beyond cavil, while the technique was so good that most of these saronged natives would be most profitable acquisitions in any London laboratory. As nurses and ward attendants they are equally good, though they lack firmness and are not usually so sympathetic as European nurses are. On the other hand, they

Balik Papan: A Street in the Native Kampong.

are very clean, neat, and precise, and can be depended on. One of the old mandoers who served the old hospital very faithfully for many years was introduced to me. He is a venerable old gentleman with one hand paralysed, so that he is unable to do any work, but he is kept on pension and acts as a sort of mentor to the others.

The hospital was built some two years ago and covers an area of some 20 acres in extent. It has its own water supply from a good artesian well, but is also connected with the main water supply of the town. Ice is supplied from the company's ice factory, which is the largest east of Suez. The supply of drugs is very good, and only the best is obtained. Thus quinine is supplied in one-gramme tablets, which considerably facilitates dispensing, in 100-tablet bottles. Dr. Horst has arranged for the pavilions to be mosquito- and fly-proof, and the work is now proceeding. Already the infectious pavilion is absolutely mosquito-proof, all the openings being covered with fine meshed mosquito wire and the double doors having the same protection. All the pavilions impress the visitor favourably. The only objection to be taken to them is the state of the floor. This is everywhere of concrete, which does not do well in a tropical hospital: it is only a year or two old and is already broken, splintered, and much excavated. Later on the flooring will be replaced by tiles or mosaic. This is specially necessary in the operating room and in the corridors.

The corridors are open to the air on both sides, but covered over, and much resemble those at Eppendorf in general appearance. Their pavements are slightly raised above the level of the ground. The operating theatre is a finely lighted and ventilated apartment, almost devoid of furniture, and from many points of view an ideal operating room, although it misses all the accessories and complications of a European operating room. The table is placed in the middle of the room, and is simple in construction and strong, without being at all luxurious or highly beautiful to look at. The walls are concreted and the floors of cement, with a slight slope for flushing. The room is provided with electric light, as are all the other rooms in the hospital, the institution being served from the central electric station, which is capable of supplying a town the size of Manchester! Next to the operating theatre is the sterilising room, which contains a good spirit steriliser, a steam steriliser, and an electrically heated steriliser; the first is most commonly used and is quite sufficient for the needs of the institution. On the other side of the entrance lobby to the operating theatre is the instrument room, which is remarkable in containing a special instrument cabinet. This is an air-tight glass case, which is furnished with electric lamps that are kept burning day and night to dry the air. Owing to the humidity of the atmosphere it has been found exceedingly difficult to keep the instruments, especially the more delicate ones, free from rust. This method seems to be the only one that answers well, but even when it is used the cystoscopes and more complicated instruments need to be continually overhauled and oiled. Rubber goods can only be preserved in water here, and enema tubes, drainage tubes, and rubber appliances generally are kept immersed in big jars of slightly alkaline water.

## Figures and Tables

**Figure f1:**
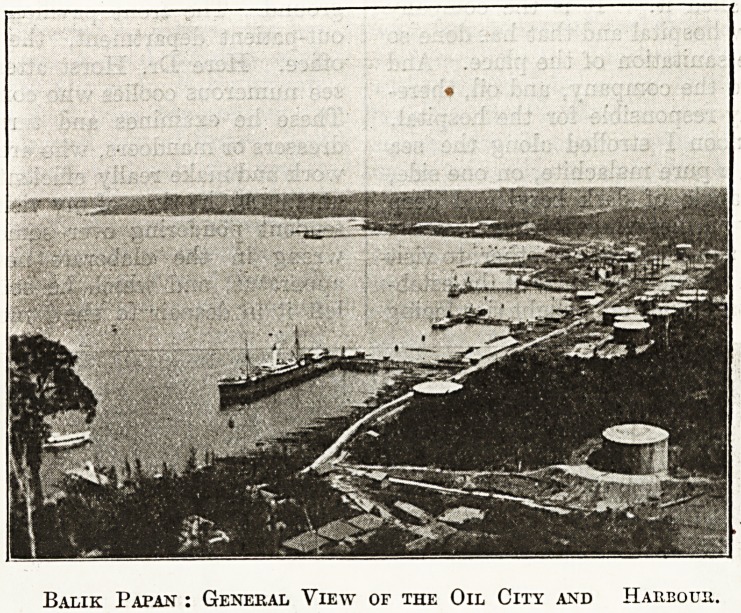


**Figure f2:**
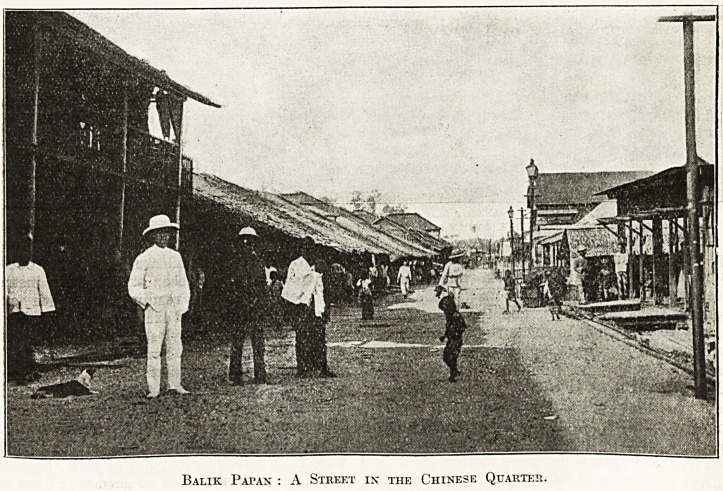


**Figure f3:**